# Cannabis-Induced Diffuse Alveolar Haemorrhage: A Case Report From the Intensive Care Unit

**DOI:** 10.7759/cureus.101619

**Published:** 2026-01-15

**Authors:** Núria Condé Pinto, Rita Xavier, Marta Pereira, Filipa Guimarães, Sara Castelo Branco

**Affiliations:** 1 Internal Medicine, Unidade Local de Saúde do Médio Ave, Vila Nova de Famalicão, PRT; 2 Intensive Medicine, Unidade Local de Saúde de Matosinhos, Porto, PRT

**Keywords:** acute respiratory distress syndrome (ards), cannabis use, diffuse alveolar haemorrhage, hypoxic respiratory failure, invasive mechanical ventilation

## Abstract

Diffuse alveolar haemorrhage (DAH) is a syndrome characterised by acute dyspnea, respiratory failure, diffuse alveolar infiltrates, haemoptysis, and anemia, which can be fatal without prompt treatment.

We present the case of a 26-year-old man with a clinical presentation of DAH and cannabis consumption. After investigation, other aetiologies were ruled out, suggesting cannabis use associated with a viral respiratory infection as the most probable cause.

Cannabis consumption has been previously documented in the literature as a potential trigger for DAH. Although rare, the clinical severity of this condition and the rising prevalence of cannabis use highlight the importance of raising awareness regarding this potential causal relationship.

## Introduction

Diffuse alveolar haemorrhage (DAH) is a syndrome that should be suspected in patients presenting with acute dyspnoea progressing to respiratory failure, diffuse alveolar infiltrates, and often haemoptysis and anaemia. However, clinical evolution may vary depending on the underlying cause, which can sometimes be sub-acute and present with nonspecific symptoms [1].

The mechanism by which haemorrhage occurs depends on the underlying aetiology, but in general, it involves alveolar circulatory dysfunction.

It is most commonly associated with vasculitis, either primary or secondary to systemic diseases or linked to conditions such as transplant rejection. Other mechanisms include bland pulmonary haemorrhage due to red blood cell extravasation without vascular inflammation, as seen in conditions such as coagulopathies, endocarditis, and mitral stenosis, and direct lesion to the vessels, as observed in acute respiratory distress syndrome (ARDS) and radiation. Some diseases, such as systemic lupus erythematosus, are associated with more than one mechanism [2].

Drug toxicity is also a cause of direct vessel lesion, with cocaine being the primary cause. However, other drugs, such as cannabinoids, have also been reported as a potential aetiology due to mechanisms such as increased intralveolar pressure related to deep inhalation [3].

Although rare, DAH constitutes a medical emergency due to potential massive bleeding and respiratory failure, which demands rapid diagnostic work-up and prompt treatment initiation. As a result, along with diagnosing DAH, elucidating its aetiology is also crucial, although sometimes challenging. Numerous conditions are associated with this clinical presentation, emphasising the importance of a proper anamnesis and physical examination to provide clues that can direct further investigation, which includes immune and toxicological studies, imaging, cardiac evaluation, and biopsies. Bronchoscopy should be performed as soon as possible to confirm the diagnosis, which is corroborated by a bloody bronchoalveolar lavage that does not lighten in serial samples, and to collect samples [4,5].

Treatment has two main goals: supportive care, including oxygen therapy, mechanical ventilation, and bleeding suppression, and control of the underlying disease, with, among others, immunosuppressants. Despite treatment, prognosis remains limited, with high mortality rates, especially in patients who progress to ventilatory failure and ARDS, where rates exceeding 50% have been reported [5,6]. We present this case of DAH due to its rare aetiology.

## Case presentation

We present the case of a 26-year-old man, employed as an agriculture worker, with a known history of cigarette smoking but no other significant medical history or chronic medication use.

He presented to the hospital with a four-day history of myalgia, asthenia, abdominal pain, nausea, and a dry cough. On admission, he had a fever (40ºC) and pharyngeal hyperaemia. Pulmonary auscultation showed no abnormalities, and respiratory insufficiency was ruled out. Laboratory analysis revealed mild anaemia, thrombocytopenia, lymphopenia, elevated C-reactive protein (CRP), and mild hepatocellular injury, with normal kidney function. Tests for respiratory viruses were negative (Table 1).

**Table 1:** Initial analytical results CRP: C-reactive protein, ALT: alanine aminotransferase, AST: aspartate aminotransferase, SARS-CoV-2: severe acute respiratory syndrome coronavirus 2.

Test	Result	Normal range
Haemoglobin (g/dL)	11.9	13.0–18.0
Leukocytes (10^3^/µL)	6.2	4.0–11.0
Lymphocytes (10^3^/µL)	0.28	1–4.8
Platelets (10^3^/µL)	108	150–400
Creatinine (mg/dL)	0.89	0.72–1.25
Urea (mg/dL)	46	17.9–55.0
Sodium (mEq/L)	141	136–145
Potassium (mEq/L)	4.2	3.5–5.1
CRP (mg/dL)	15.5	0–0.5
ALT (U/L)	121	0–55
AST (U/L)	76	0–34
Respiratory virus panel (antigen)		
Influenza A	Negative	-
Influenza B	Negative	-
SARS-CoV2	Negative	-
Respiratory syncytial virus	Negative	-

**Figure 1:**
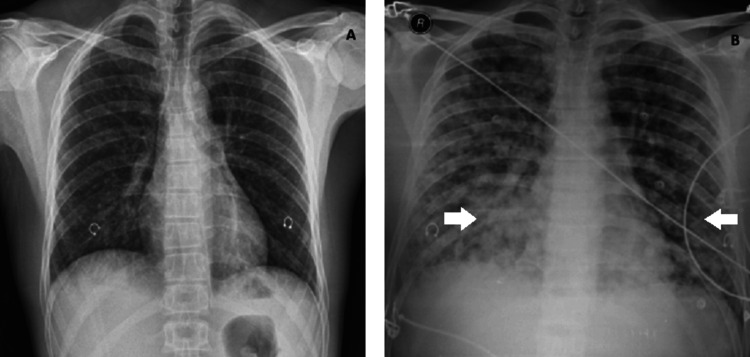
Evolution in chest radiograph at presentation, showing a normal image (A) and progression with bilateral diffuse pulmonary infiltrates (B)

A chest radiograph showed mild enhancement of the bronchovascular network, without evidence of pulmonary infiltrates (Figure 1A). He was kept under observation and discharged with symptomatic treatment advice. However, three hours later, he returned to the hospital with worsening symptoms, including persistent fever, dyspnoea at rest, haemoptoic sputum, and light-headedness. At this point, he had developed respiratory failure requiring invasive mechanical ventilation. Bloody secretions were observed during aspiration. A chest radiograph was repeated and revealed extensive bilateral diffuse pulmonary infiltrates (Figure 1B).

He remained haemodynamically stable without hyperlactacidaemia. Empirical antibiotic therapy with amoxicillin-clavulanic acid and azithromycin was initiated due to suspected pneumonia. Further blood samples showed worsening anaemia and thrombocytopenia, along with evidence of rhabdomyolysis (Table 2). Blood and urine cultures were collected. Subsequently, he was transferred to our intensive care unit (ICU).

**Table 2:** Subsequent analytical study after clinical worsening CRP: C-reactive protein, ALT: alanine aminotransferase, AST: aspartate aminotransferase, GGT: gamma-glutamyl transferase, ALP: alkaline phosphatase, CK: creatine kinase, HIV: human immunodeficiency virus.

Test	Result	Normal range
Haemoglobin (g/dL)	10.3	13.0–18.0
Leukocytes (10^3^/µL)	6.2	4.0–11.0
Lymphocytes (10^3^/µL)	0.1	1-4.8
Platelets (10^3^/µL)	89	150–400
CRP (mg/dL)	18.8	0–0.5
ALT (U/L)	119	0–55
AST (U/L)	84	0–34
GGT (U/L)	45	0–55
ALP (U/L)	51	40–150
Bilirubin	0.7	0.2–1.2
Creatinine kinase (U/L)	900	130–200
Myoglobin (U/L)	600	7–105
Creatinine (mg/dL)	0.81	0.7–1.25
Urea (mg/dL)	46	17.9–55.0
Sodium (mEq/L)	141	136–145
Potassium (mEq/L)	4.2	3.5–5.1
Microbiological study		
Pneumococcal antigen	Negative	-
Legionella antigen	Negative	-
Human immunodeficiency virus antibody (UI/mL)	0.05	<1.0

During his ICU stay, the patient’s management focused on clinical stabilisation and aetiological investigation. Initially, obtaining a comprehensive clinical history was not possible, as the patient was intubated and attempts to communicate with significant relatives were unsuccessful. On initial objective examination, a pyknic biotype and missing teeth were noted, with no skin lesions, organomegaly, or lymphadenopathy identified. Abundant bloody secretions were suctioned.

**Figure 2:**
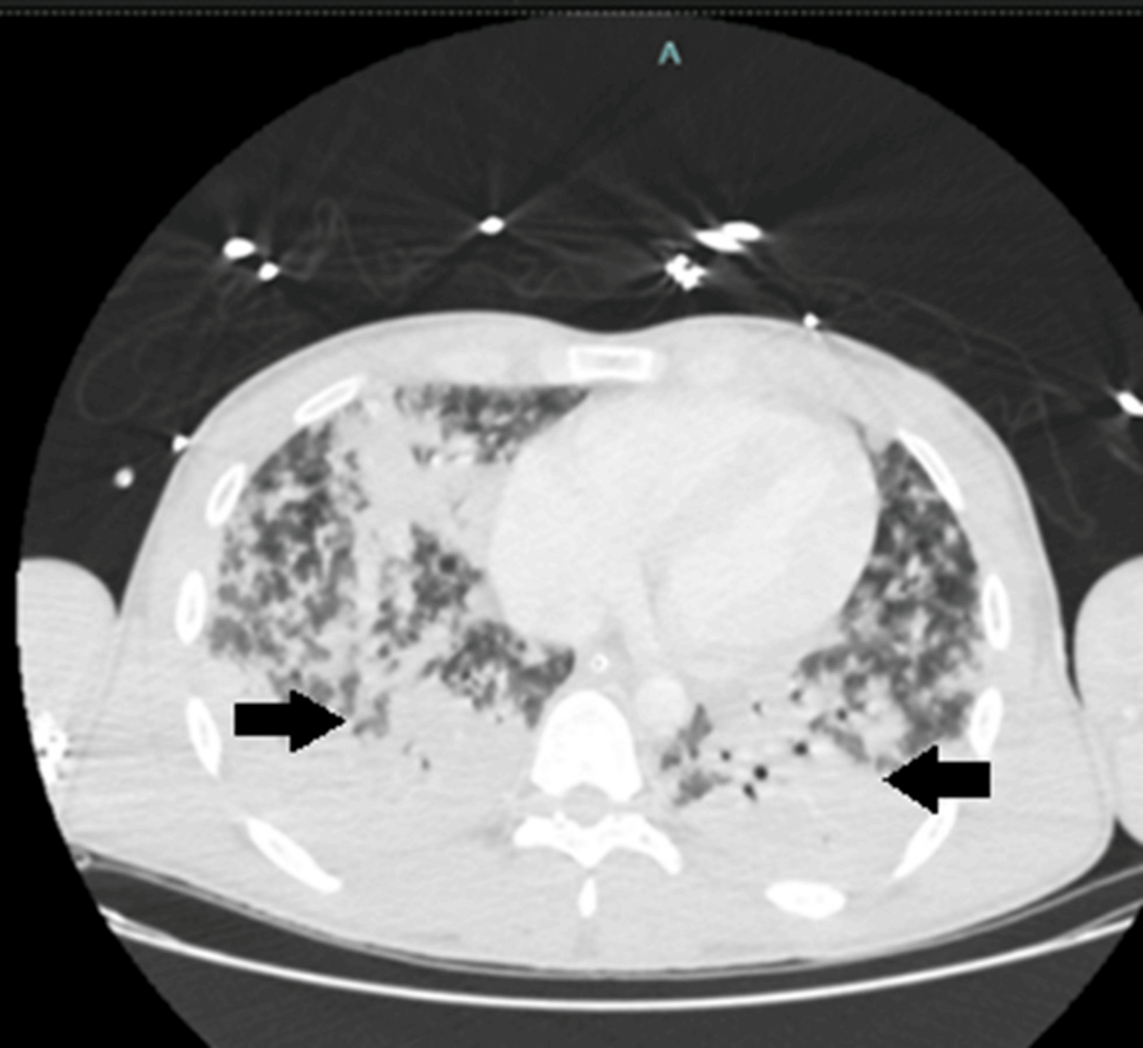
Thoracic CT showing bilateral lung consolidations

A computed tomography (CT) angiography of the chest was performed (Figure 2), revealing parenchymal consolidations with air bronchograms in the inferior right lobe and centimetre-sized confluent multifocal parenchymal consolidations in the remaining lung segments, along with mild pericardial and bilateral pleural effusion.

**Figure 3:**
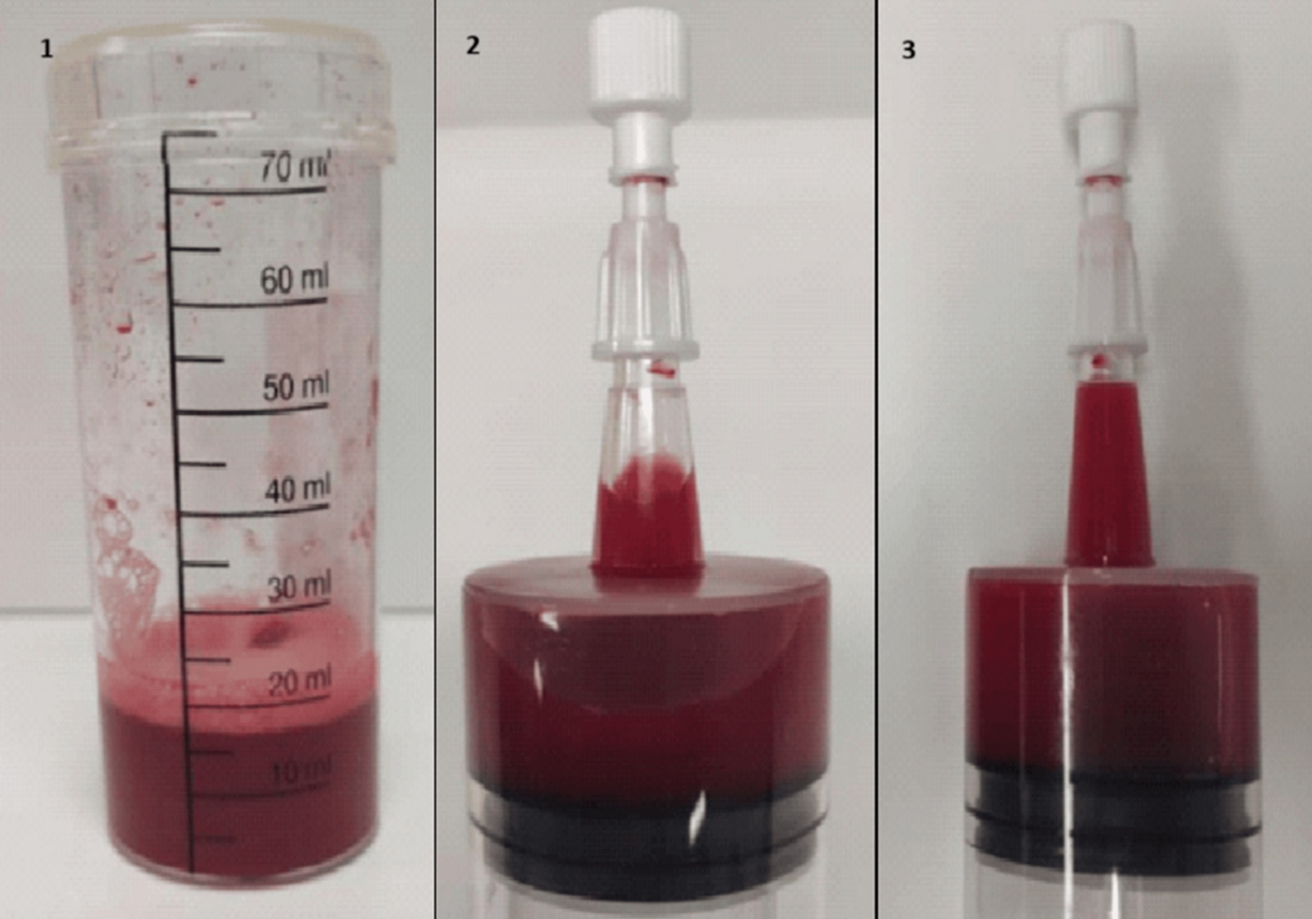
Consecutive BAL samples BAL: bronchoalveolar lavage.

At this point, DAH was suspected, prompting bronchoscopy for further investigation, during which three consecutive haematic samples were collected, supporting this suspicion (Figure 3). The examination revealed diffuse hyperaemia of the bronchial tree, with abundant bloody secretions but no active haemorrhage.

Therefore, an aetiological study was conducted considering the most probable causes of alveolar haemorrhage. Samples were sent for microbiological, cytological, and immunological analysis. Immunoserological assays and urine sediment examination were performed to investigate the possibility of an immune-mediated vasculitic process, while specific microbiological studies, including those for zoonotic agents, were undertaken due to the patient’s agricultural background. Due to the possibility of a cardiac aetiology, such as endocarditis or mitral valve dysfunction, a transthoracic echocardiogram was performed and showed no pathological findings. Screening for drug abuse was conducted, given its association with DAH. Upon admission to the ICU, antibiotic therapy was switched to azithromycin plus doxycycline due to the suspicion of zoonotic agents.

Ventilation was set in Pressure-Regulated Volume Control (PRVC) mode, with a positive end-expiratory pressure (PEEP) of 8 cmH₂O and a tidal volume of 500 mL. In the first 48 hours, respiratory function improved, allowing a reduction in oxygen supplementation from 40 to 21%, with a favourable P/F ratio (>350), and sedation adjustment to achieve a RASS score of -2. Haemoglobin decreased to 7 g/dL, while platelet count dropped to 56 × 10^3^ µL, motivating transfusion of one unit of erythrocytes and administration of tranexamic and aminocaproic acid.

Further study showed an elevated erythrocyte sedimentation rate and a positive drug screen for cannabinoids and opioids. The bronchoalveolar lavage evidenced blood and neutrophils. The complete results are presented in Table 3.

**Table 3:** Initial aetiological study in the ICU HPF: High Power Field; PCR: Polymerase Chain ReactionESR: erythrocyte sedimentation rate, C3: complement component 3, C4: complement component 4, Anti-ds-DNA: anti–double-stranded DNA antibody, ENA: extractable nuclear antigen, PR3: proteinase 3, MPO: myeloperoxidase, HPF: high-power field, PCR: polymerase chain reaction.

Test	Result	Normal range
Immune study		
Erythrocyte sedimentation rate (mm/h)	73	<20
C3 (mg/dL)	103.0	82–185
C4 (mg/dL)	19.0	15–53
Antinuclear antibodies	Negative	-
Anti-ds-DNA	Negative	-
ENA antibodies	Negative	-
Anti-PR3 antibody (UI/mL)	<0.2	<2
Anti-MPO antibody (UI/mL)	<0.8	<5
Anti-glomerular basement antibody (UI/mL)	<0.8	<7
Rheumatoid factor (UI/mL)	<20	<30
Eosinophils (10^3^/µL)	0.10	0.0–0.7
Urine		
Blood (mg/dL)	0.1	<0.003
Leucocytes (uL)	0	<25
Proteins (mg/dL)	10	<10
Nitrites (mg/dL)	Negative	-
Drugs		
Cocaine	Negative	-
Benzodiazepines	Negative	-
Cannabinoids	Positive	-
Opioids	Positive	-
Bronchoalveolar lavage		
Epithelial cells	<10	-
Blood	Positive	-
Neutrophils (HPF)	>25	-
Microbiology (molecular biology-PCR)		
Influenza A	Negative	-
Influenza B	Negative	-
Parainfluenza	Negative	-
Adenovirus	Negative	-
Respiratory syncytial virus	Negative	-
Metapneumovirus	Negative	-
Legionella	Negative	-
*Chlamydophila pneumoniae*	Negative	-
*Mycoplasma pneumoniae*	Negative	-

On the third day of hospitalization, the patient presented with clinical deterioration characterized by worsening respiratory insufficiency with a P/F ratio <200, requiring an increase in oxygen supplementation to FiO₂ 60%, along with bronchospasm, cough, and significant haemorrhage. Sedation and curarization were required, with a BIS target of 40-60. Due to the absence of an evident aetiology but significant clinical deterioration resembling an ARDS-like evolution, corticosteroid therapy with methylprednisolone 1 mg/kg/day was initiated. Subsequently, the patient showed marked global improvement, leading to successful extubation on day five and a reduction in oxygen supplementation to 1 L/min via nasal cannula.

**Figure 4:**
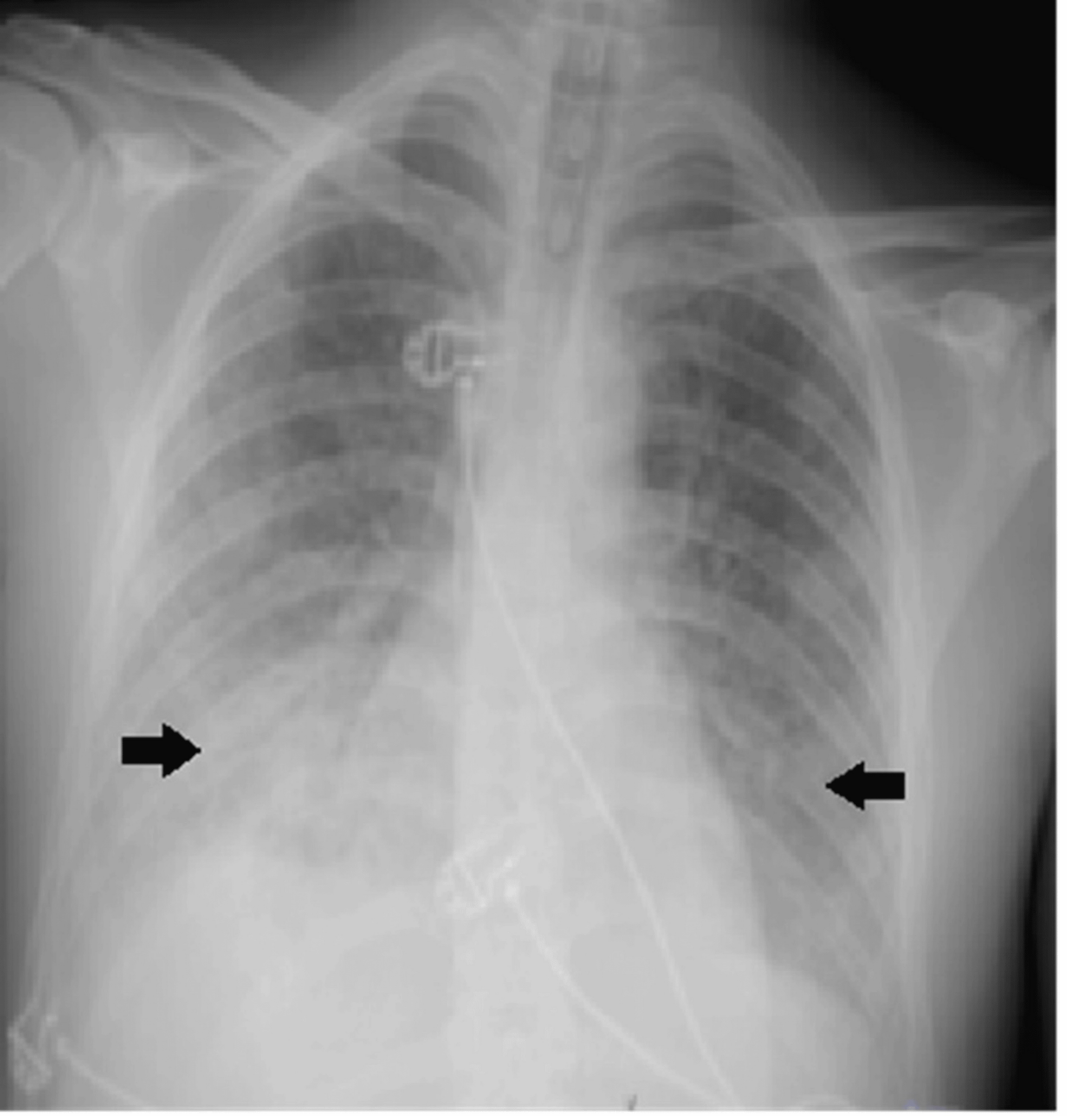
Control chest radiograph showing improvement in lung infiltrates

After waking up, he confirmed the described history and denied any other symptoms, chronic or acute. He also admitted to consuming illicit drugs, cannabinoids by blunt smoke and previously inhaled cocaine. He denied recent travel or the use of pesticides at work. Repeat radiography showed a reduction of pulmonary infiltrates (Figure 4).

There were also analytical improvements, with resolution of lymphopenia and reduced CRP (2.5 mg/dL). Anaemia improved to 10 g/dL after a second transfusion, and platelet count normalized. Therefore, after sustained improvement, the patient was transferred to a hospital ward, with the suggestion of continuing doxycycline for a total of seven days. Later, the bronchoalveolar lavage immunophenotypic analysis showed a predominance of neutrophils (89%). Culture and additional microbiological tests were negative (Table 4).

**Table 4:** Aetiological study - further results CD4: cluster of differentiation 4, CD8: cluster of differentiation 8, CD103: cluster of differentiation 103, IgG: immunoglobulin G, IgM: immunoglobulin M, PCR: polymerase chain reaction.

Test	Result	Normal range
Bronchoalveolar lavage		
Immunophenotyping		
B lymphocytes (%)	0.87	-
T lymphocytes (%)	3.26	-
CD4+ lymphocytes (%)	2.22	-
CD4+CD103+ (%)	0.78	-
CD4+CD103- (%)	0.01	-
CD8+ lymphocytes (%)	0.25	-
Neutrophils (%)	89.18	-
Macrophages (%)	4.2	-
Eosinophils (%)	2.0	-
Microbiology		
Bacterial and mycobacterial culture	Negative	-
Blood		
Bacterial culture	Negative	-
Mycobacterial culture	Negative	-
Coxiella burnetii – serology (title)		
Phase I IgG	<200	<200
Phase I IgM	<50	<50
Phase II IgG	<200	<200
Phase II IgM	<50	<50
Leptospira – molecular biology (PCR)	Negative	-
Hantavirus – serology		
IgM	<16	<16
IgG	<32	<32
Urine		
Leptospira – molecular biology (PCR)	Negative	-

By this time, we postulate that the most likely mechanism of disease was a viral infection associated with drug-induced alveolar haemorrhage.

## Discussion

This case is an example of diffuse alveolar haemorrhage and illustrates the diagnostic and therapeutic challenges related to this pathology. The impossibility of obtaining a proper anamnesis was an initial limitation, only overcome by a systematic assessment based on the clinical presentation, which guided the diagnostic process and appropriate supportive treatment.

Regarding the aetiology, some questions remain to be clarified. Going back to the beginning of the episode, there was a clinical picture suggestive of an acute infection, presumably viral, taking into account the symptoms and the initial analytical study. However, no agent was identified in the different samples collected, leaving the possibility of an untested agent or that the infection was not present.

As for pancytopenia, it seems to be justified by infection or inflammation and the acute haemorrhagic context, corroborated by its recovery after clinical improvement and blood transfusion.

On the other hand, there was a history of drug abuse, namely past inhaled cocaine and current smoked cannabinoids, with urine testing being positive for the latter. Cocaine-associated lung disease, namely DAH, is already well described, but cannabis has also been suggested in this lesion mechanism [3]. There was also a positive screen for opioids, which appears less valid in this context since fentanyl was being used as the analgesic drug at the time of urine collection. There are also several potential toxic materials associated with the manufacture of cannabis and cocaine preparations.

The BAL interpretation is also challenging, as it is not as favourable to viral infection, but can be justified by toxic lesions. The clinical evolution and test results disfavour other diagnoses, such as immune-mediated diseases and cardiogenic aetiology.

Acute cannabis-related physiological effects are described in the literature, particularly neuropsychiatric effects such as anxiety and delirium, conjunctival injection, tachycardia, dry mouth, cough, and asthma exacerbation. Cannabis-induced alveolar haemorrhage has already, although very rarely, but increasingly, been reported. The mechanism is not fully understood, but it can be theorized as a combination of direct inflammation of the alveoli associated with increased intralveolar pressure due to deep inhalation and the Valsalva manoeuvre. This is also in line with pneumothorax and pneumomediastinum associated with cannabis smoking, already described [3,7-9]. It has also been suggested that cannabinoids may impair alveolar macrophage function, increasing infection risk [10].

Therefore, our interpretation is of a possible synergistic mechanism of lung injury caused by exposure to drugs, acutely exacerbated in the context of an unidentified infection, which may promote inflammation and an increase in intralveolar pressure due to cough, with a critical evolution.

Regarding supportive treatment, antibiotics were initiated because of the severity and lack of clinical history, but they were probably not needed. The use of generalized corticosteroids in these patients is controversial [5]. In this case, they were determinant for clinical improvement by reducing local inflammation and preventing perpetuation of the mechanism of endothelial injury, regardless of the absence of underlying autoimmune disease.

## Conclusions

DAH is a rare but serious and potentially fatal clinical entity. It should therefore be addressed within the scope of a medical emergency, with the rapid institution of supportive treatment and timely diagnostic investigation regarding toxic causes, where some of the constituents are unknown.

Cannabis-associated diffuse alveolar haemorrhage is a very rare condition described in the literature, which may be taken into account in patients with this clinical presentation and history of cannabis consumption. As a diagnosis of exclusion, more common causes should always be evaluated and excluded.
